# Diseases of the Heart and Circulation

**Published:** 1903-12-26

**Authors:** 


### Diseases of the Heart and Circulation.

**Stokes-Adams Disease.**—Many interesting cases of this rare disease are recorded by Osler,[1] together with a discussion of its nature and treatment. The disease is a clinical condition characterised by (1) a disturbance in the automatic mechanism of the heart —true bradycardia, hemisystole (false bradycardia) and allorythmia; (2) nervous symptoms—vertigo, syncope, pseudo-apoplexy and epileptiform attacks; and (3) secondary symptoms — Cheyne-Stokes breathing, cardiac asthma, angina pectoris and the vaso-motor accompaniment of profound heart shock. The post-mortem changes are not constant, in a few cases coarse lesions of the nervous system have been found, in a few no lesions whatever, but generally arterio sclerosis is present. A slow pulse is found in some few individuals as a physiological condition; in organic disease of the nervous system, in toxic conditions, *e.g.* diphtheria and typhoid fever, digitalis and tobacco poisoning, and in uræmia and jaundice, and in cardiac and cardio-vascular lesions. Stokes-Adams disease is a symptom-complex, not one with constant anatomical lesions or a uniform etiology. The cases may be divided into three categories. (1) *Post-febrile group*—following some acute affection, such as typhoid fever, pneumonia or rheumatism, bradycardia may occur with vertigo, syncope, or epileptiform seizures. This form is the most hopeful, though the first attack may prove fatal. (2) *Neuritic group*—generally with coarse lesions of the nervous system, most often extrinsic. (3) *Arterio-sclerotic group*—comprising by far the majority of cases which have been recorded. The diagnosis in the severe form is usually simple, and is only difficult in cases of aggravated neurasthenia associated with bradycardia and vertigo. The cardio-vascular features are of great interest. The bradycardia is usually a true one, *i.e.*, the heart beats and pulse beats are of equal rate. In a few cases this true bradycardia alternated with a false one, *i e.*, one in which the pulse was slow because only every alternate beat reached the wrist. The pulse is usually full, strong and regular; the infrequency seems to be due to prolongation of the diastole, the systole being sharp and quick. The rate may be anything from 40 down to 12 per minute. In false bradycardia the abortive beats may sometimes be seen and felt at the apex, more often they may be heard, they may be alternate with the beats reaching the wrist, or much more frequent, *e.g.*, in one case the radial pulse was 19 to the minute, with 74 palpable visible and audible cardiac beats. One of the most remarkable features in some cases is a state of cardiac arrest—which may be so long as 35 seconds—at the commencement of the attack. The patient usually seeks relief for nervous features—which may be either vertigo, syncope, pseudo-apoplexy (*i.e.*, sudden coma with loud stertorous breathing and deeply congested face), or epileptic seizures sometimes resembling true epilepsy. A characteristic feature of all of these attacks is their frequently recurring character. The pathology is very obscure. The cerebral features are probably due to a transitory anæmia of the nerve centres. But we do not know the explanation of the cardiac features of the attack, *e.g.* to every perfect four or five abortive systoles, extreme slowness of the heart, non-propagation of the auricular rhythm to the ventricle, and the periods of transient arrest of the heart. The outlook is bad in all forms and in all cases. Bradycardia may last for many years with good health, but once the severer nervous symptoms have begun there is little prospect of complete relief. Sudden death is the most common. In younger patients iodide may be given, and with high tension nitrites are indicated. In the senile form food may be restricted and the bowels kept open. We have no remedy which will accelerate a permanently slow heart. For the syncope nitrite of amyl and ammonia may be used.

[1] Lancet, Aug. 22, 1903.

*(To be continued.)*

